# Emergence of Metastable State Dynamics in Interconnected Cortical Networks with Propagation Delays

**DOI:** 10.1371/journal.pcbi.1003304

**Published:** 2013-10-24

**Authors:** Katrina M. Kutchko, Flavio Fröhlich

**Affiliations:** 1 Department of Psychiatry, University of North Carolina at Chapel Hill, Chapel Hill, North Carolina, United States of America; 2 Curriculum in Bioinformatics and Computational Biology, University of North Carolina at Chapel Hill, Chapel Hill, North Carolina, United States of America; 3 Department of Cell Biology and Physiology, University of North Carolina at Chapel Hill, Chapel Hill, North Carolina, United States of America; 4 Department of Biomedical Engineering, University of North Carolina at Chapel Hill, Chapel Hill North Carolina, United States of America; 5 Neuroscience Center, University of North Carolina at Chapel Hill, Chapel Hill, North Carolina, United States of America; Institut de Neurosciences des Systèmes, France

## Abstract

The importance of the large number of thin-diameter and unmyelinated axons that connect different cortical areas is unknown. The pronounced propagation delays in these axons may prevent synchronization of cortical networks and therefore hinder efficient information integration and processing. Yet, such global information integration across cortical areas is vital for higher cognitive function. We hypothesized that delays in communication between cortical areas can disrupt synchronization and therefore enhance the set of activity trajectories and computations interconnected networks can perform. To evaluate this hypothesis, we studied the effect of long-range cortical projections with propagation delays in interconnected large-scale cortical networks that exhibited spontaneous rhythmic activity. Long-range connections with delays caused the emergence of metastable, spatio-temporally distinct activity states between which the networks spontaneously transitioned. Interestingly, the observed activity patterns correspond to macroscopic network dynamics such as globally synchronized activity, propagating wave fronts, and spiral waves that have been previously observed in neurophysiological recordings from humans and animal models. Transient perturbations with simulated transcranial alternating current stimulation (tACS) confirmed the multistability of the interconnected networks by switching the networks between these metastable states. Our model thus proposes that slower long-range connections enrich the landscape of activity states and represent a parsimonious mechanism for the emergence of multistability in cortical networks. These results further provide a mechanistic link between the known deficits in connectivity and cortical state dynamics in neuropsychiatric illnesses such as schizophrenia and autism, as well as suggest non-invasive brain stimulation as an effective treatment for these illnesses.

## Author Summary

The brain mediates behavior by orchestrating the activity of billions of neurons that communicate with each other through electric impulses. The transmission of these action potentials is surprisingly slow for a large fraction of these connections. Given the importance of precise timing of neuronal activity, the function of these slow connections has remained a puzzle. We here used computer simulations to investigate how slow connection speeds alter the overall activity patterns of two brain networks. We found that these connections enable the interconnected networks to generate distinct activity patterns such as different types of waves of electric activity. Our results therefore suggest that the slow transmission of electric impulses in the brain is not a “design flaw” but rather plays an important role in enabling the brain to generate a richer set of activity patterns. The ability of the brain to switch between different activity states is crucial to normal cognition, and abnormalities in switching behavior are associated with cognitive symptoms in psychiatric disorders such as schizophrenia and autism. It is therefore promising that we were able to control transitions between different activity states with non-invasive brain stimulation in our simulations, suggesting a novel approach to the treatment of these illnesses.

## Introduction

Cognition emerges from the organized temporal structure of electric activity in large, interconnected cortical networks [1]–[3]. The network topology is a key determinant of the types of macroscopic activity patterns a network can generate [4]–[11]. Understanding this structure-function relationship provides important insight not only into normal brain function but also into the mechanistic basis of psychiatric illnesses such as schizophrenia and autism that likely represent “connectivity disorders” [12]–[15]. These connectivity disorders are associated with both structural and functional impairments in connectivity [16]–[19]. Consequently, an understanding of the relationship between network topology and dynamics will facilitate the development of new treatment modalities that counteract dysfunctional network connectivity in psychiatric illnesses.

Systematic parameterization of network topology in computational models has demonstrated that connections between random pairs of distant, excitatory neurons within a network enhance temporal synchronization, whereas predominantly local connectivity between neighboring excitatory neurons facilitates macroscopic activity patterns such as oscillations and planar and spiral waves that propagate through the network [20]–[22]. However, individual cortical networks seldom act in isolation because of their interconnectivity with other networks by means of long-range projections (LRPs). Most studies of interconnected networks have focused on how networks synchronize via fast LRPs, with the exception of recent theoretical work that highlights the additional complexity and computational abilities of networks that include physiological delays [23]–[25].

Mathematical studies of the effects of delays on coupled oscillators have predicted diverse results as a consequence of delays. Foundational papers have found that delays between coupled systems produce stability under certain parameters [26], including stability of synchronization in systems of coupled neurons [27]. Delays have also been shown to generate bifurcations and multistability in coupled oscillator systems [28], [29] and neural loops [30], and to give rise to bifurcations and instability in neural field models [31], [32]. Recently, multistability as a result of delays was found in a Hopfield neural network model [33]. This presence of multistability in such abstract models of neurons and networks of neurons suggests that propagation delays promote multistability. In order to bridge the gap between abstract, theoretical models and biology, we built a large-scale, detailed model of two interconnected cortical networks. The spiking neuron models used in our study accurately reflected the subthreshold dynamics of real neurons and were subject to noise injections that mimicked the stochastic nature of neuronal signaling. With this model, we examined the functional role of the estimated fifty percent of connecting axons with long propagation delays as a consequence of small axonal diameter or a lack of myelination [34]–[36].

We hypothesized that slower long-range projections may enrich overall network activity by counteracting and disrupting the intrinsic, spontaneous dynamics of individual networks. According to our hypothesis, slower projections provide perturbations that are ill-timed to synchronize networks and therefore enable different activity trajectories that individual networks are unable to generate. To test this hypothesis, we used large-scale computer simulations to ask what role long-range projections with propagation delays may play in organizing the overall dynamics of two interconnected cortical networks with intrinsic spontaneous dynamics similar to isolated cortical networks *in vivo* [37], [38]. We found that such projections greatly enlarge the repertoire of macroscopic activity patterns in comparison to the networks without propagation delays and that these patterns corresponded to metastable activity states. The interconnected networks spontaneously transitioned between these states. We then evaluated non-invasive brain stimulation (transcranial Alternating Current stimulation, tACS) [39]–[41] as a tool to manipulate these dynamics and found that both in-phase and anti-phase tACS induced and guided state transitions. These findings are of broad translational importance since transitions between metastable macroscopic activity states have recently emerged as a fundamental organizational principle of cortical activity, the dynamics of which are impaired in neuropsychiatric disorders [42], [43]. Our results therefore suggest a novel mechanism of multistability in cortex and a therapeutic modality with which to manipulate cortical dynamics.

## Results

### Zero Time-Lag Synchronization by LRPs without Propagation Delays

**Figure 1:**
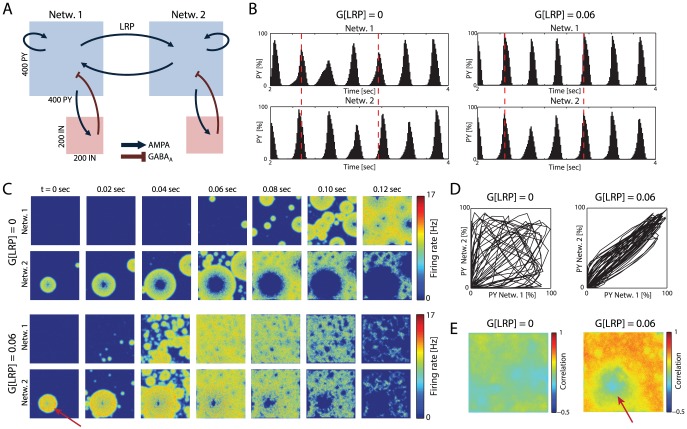
Long-range projections synchronize cortical areas in large-scale computational model. (A) Network model. Each network consists of 160,000 excitatory pyramidal neurons (PYs) and 40,000 inhibitory interneurons (INs). Synaptic connectivity: PY-PY, IN-PY: AMPA synapses; IN-PY: GABA_A_ synapses. PYs in both networks are mutually connected by AMPAergic long-range projections (LRPs). (B) PY activity in each network. Left: No LRPs. Right: With LRPs. (C) Time snapshots of binned PY firing rates. Without (top) and with LRPs (bottom), Network 2 fires before Network 1; with LRPs, UP states are synchronized. Onset site remains the same (red arrow). Color represents instantaneous firing rate. (D) Phase space plots comparing the percentage of PYs firing in Network 1 with the percentage firing in Network 2. Trajectory close to unity line indicates synchronization of PY activity across networks in the presence of LRPs (right). (E) Correlation coefficients between homologous PYs across networks. Area of reduced correlation coefficients corresponds to the region of initiation of UP states in Network 2 (arrow).

To understand the effect of long-range projections (LRPs) on the dynamics of two interconnected cortical networks, we built a large-scale computational model of two networks connected by LRPs (Fig. 1A) where each network consisted of a two-dimensional sheet of excitatory pyramidal cells (400×400 PYs) and a matched sheet of inhibitory interneurons (200×200 INs). The synaptic connectivity within the two excitatory-inhibitory networks was chosen to generate slow rhythmic activity in the absence of LRPs (Fig. S1A), a hallmark activity pattern of isolated cortex [37], [38], that was structured by alternating epochs of activity (UP states) and quiescence (DOWN states). As expected, adding sparse instantaneous (zero-delay) LRPs at the same synaptic strength as the local PY-PY excitation (G(LRP) = 0.06, P(local) = 99%) synchronized the activity pattern of PYs across networks (Fig. 1B: Fraction of PYs active as a function of time; left: no LRPs; right: with LRPs; see also Fig. S1A, sample PY membrane voltage traces).

Both with and without LRPs, UP states emerged as initially localized “regions of initiation” that then expanded through the local excitatory connectivity (circular patterns in Fig. 1C, time snap-shots of firing rates). In the presence of LRPs, the UP states synchronized their occurrence across the two networks (Fig. 1C, bottom; Fig. 1D: phase-plane representation, left: no LRPs; right: with LRPs), which increased the correlation of individual neurons with their homologous partner in the other network (Fig. 1E, correlation coefficients for PY membrane voltages; left: no LRPs; right: with LRPs). The region of initiation in Network 2 (arrow in 1C) corresponded to the area of low correlation (arrow in 1E) since the local connections within that network mostly contributed to that region's activity when Network 1 was in a DOWN state. The endogenous network oscillation of the two unconnected networks was only minimally altered by LRPs (spectral peak at 3.2 and 3.3 Hz for the two unconnected networks with peak power of 3.39e7 and 3.33e7, respectively; with LRPs: 3.3 Hz for both networks with 3.79e7 and 3.57e7 peak power, in arbitrary units, Fig. S1B). Therefore, LRP without propagation delays enabled the synchronization of the intrinsic network activity states without pronounced changes to the overall dynamics of the individual networks. Changing the LRP from a random pattern to a homologous configuration further enhanced inter-network synchronization (Fig. S2).

### LRPs with Delays Enable Emergence of Multiple Network States

To mimic realistic delays in action potential propagation along low-diameter and unmyelinated fibers that connect different networks, we next added physiologically plausible delays [36] to the LRPs such that presynaptic action potentials in one network led to delayed postsynaptic activity in the other network (1,2,5,10,30,and 50 msec). We ran simulations for parameterized number and strength of LRPs (P(local): 0.95, 0.97, 0.99, 0.999; G(LRP) = 0.015, 0.03, 0.06, 0.09, 0.12; 100 simulations per delay value) to evaluate the effect of delays on the overall dynamics (five simulations per parameter set with different noise values). Because the number and strength of LRPs in the human cortex is not well-characterized, we used a range of parameters to explore the spatio-temporal activity patterns that result from different LRP parameter sets. In our model, P(local) values of 99% and 99.9% resulted in approximately 30% and 3.6% of neurons having LRPs, respectively. These numbers are similar to the LRP numbers reported for murine cortex [44].

**Figure 2:**
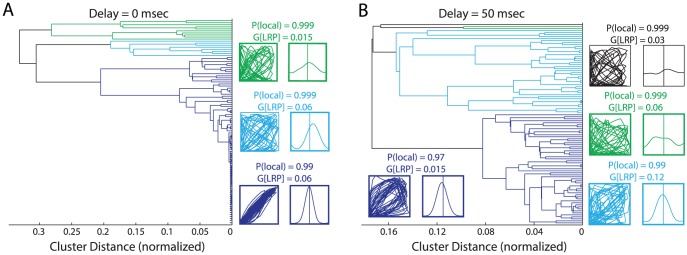
LRP delays broaden the set of interaction dynamics between the two networks. Simulations clustered by maximum cross-correlation value with linkage analysis. Phase space plots and cross-correlograms shown for all clusters (defined by 90% of full tree). Dark blue represents clusters with the greatest magnitude of cross-correlation maxima, followed by cyan, green, and black. (A) LRP delay: 0 msec. (B) LRP delay: 50 msec. Insets: Phase space plots and cross-correlograms.

We clustered the simulation outputs with linkage analysis using the peak cross-correlation value, which measures the overall synchronization of the two PY networks (dendrograms in Fig. 2A: 0 msec and Fig. 2B: 50 msec delays, respectively). In the absence of propagation delays, simulations were tightly linked, showing similarity of behavior across simulations. The majority of simulations (82%) fell into a single cluster with close to maximum synchronization index (Fig. 2A, dark blue) with only a small fraction exhibiting different behavior (8%, cyan). Thus, without delays in the LRPs, the overall network behavior was very consistent and robust. For increased delays, the relative branch lengths within each cluster became longer and fewer simulations were grouped with the most-synchronized cluster (Fig. 2B, 55% dark blue, 42% cyan, 2% green, 1% black). Therefore, in agreement with our initial hypothesis, these results demonstrate that propagation delays increase the number of different configurations the connected networks can occupy as a function of the LRP parameters.

**Figure 3:**
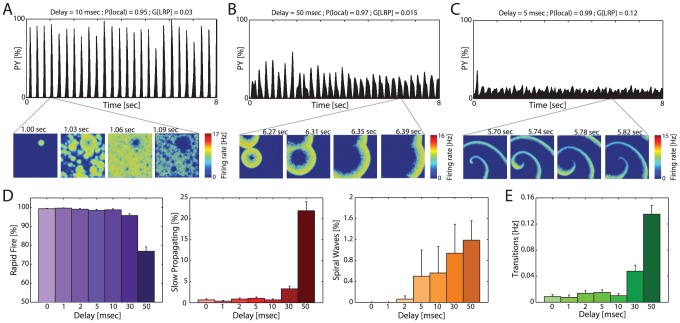
Emergent network activity states for LRP delays. (A–C) Cortical activity states characterized by different spatio-temporal activity patterns. Top: PY activity throughout simulation. Bottom: Time snapshots of PY activity. (A) Rapid fire state (RF): synchronized PY firing within a network. (B) Slow propagating state (SP): Activity originates in one or a few places and slowly traverses through the network. (C) Spiral wave state (SW): waves propagate from a central rotor in a spiral shape. (D) Percentage of time simulations spent in each state by delay, separated by network state (RF, SP, SW from left to right). (E) Frequency of state transitions by delay. All error bars represent s.e.m.

We then examined how these different synchronization patterns impacted the intrinsic dynamics within the individual networks. Indeed, inspection of the spatio-temporal activity profiles revealed the occurrence of three distinct patterns, which can be classified as network states. Typically, networks were in a rapid fire (RF) state, with most PYs in the network firing almost simultaneously and the network as a whole demonstrating slow oscillatory behavior (Fig. 3A, top: pronounced peaks correspond to network-wide UP states in PY activity pattern; bottom: consecutive time snapshots of PY firing activity; see also Movie S1). However, the addition of delays to the LRPs also supported two alternate forms of spatio-temporal dynamics: slow propagating (SP) state, with regional UP states originating in one or a few areas and slowly traversing through the local network (Fig. 3B, top: rhythmic structure is less apparent in network-wide activity profile due to lack of zero-lag synchrony within the network, bottom: initial onset of UP state morphs into a propagating, expanding wave front; see also Movie S2); and spiral wave (SW) state, with a wave originating from single (or occasionally multiple) rotor in a spiral pattern (Fig. 3C; see also Movie S3).

Next, we asked how the occurrence of these three different macroscopic network states depended on LRP delays. We found that most interconnected networks followed an RF pattern, especially for short LRP delays (Fig. 3D, left, relative percent of time spent in RF: 99.31±0.24% for 0 msec delay versus 76.88±2.26% for 50 msec, mean±s.e.m., Table S1). For longer delays, the percentage of time spent in RF decreased and SP became more prominent (Fig. 3D, middle, SP for 0 msec delay: 0.69±0.24%; 21.94±2.15% for 50 msec delay). Also, SW, which never occurred in the absence of delays, increased its relative presence with larger delays (Fig. 3D, right, 1.19±0.37%, note different scales). We then further examined if the interconnected networks stayed in one state for the entire simulation or whether they exhibited spontaneous transitions between these states. We found that, in general, the networks only remained in the same state without transitioning for short LRP delays (Fig. 3E, average transition frequencies, 0.0088±0.0031 Hz for 0 msec delay; 0.135±0.0134 Hz for 50 msec delay, see also Table S1). Therefore, longer (and thus more realistic) propagation delays increased not only the presence of other, non-RF states but also the number of transitions between states.

In order to further evaluate the robustness of this result, we also tested the effects of a distribution of delays. We ran two sets of simulations, the first with delays uniformly distributed ±20% of the mean and the second with delays uniformly distributed ±100% of the mean. Our results indicate that wider distributions resulted in fewer state transitions (Fig. S3A, top: narrow distribution, 0.0981±0.0104 Hz for 50 msec delay, bottom: wide distribution, 0.0694±0.0091 Hz for 50 msec delay, see also Table S2). Additionally, a broader distribution of delays resulted in less time spent in SP (Fig. S3B). Consequently, a wider distribution of LRP delays, which entails a greater number of shorter delays, seems to stabilize network behavior yet does not abolish multistability.

### Transitions between Metastable Spatio-Temporal Activity States

**Figure 4:**
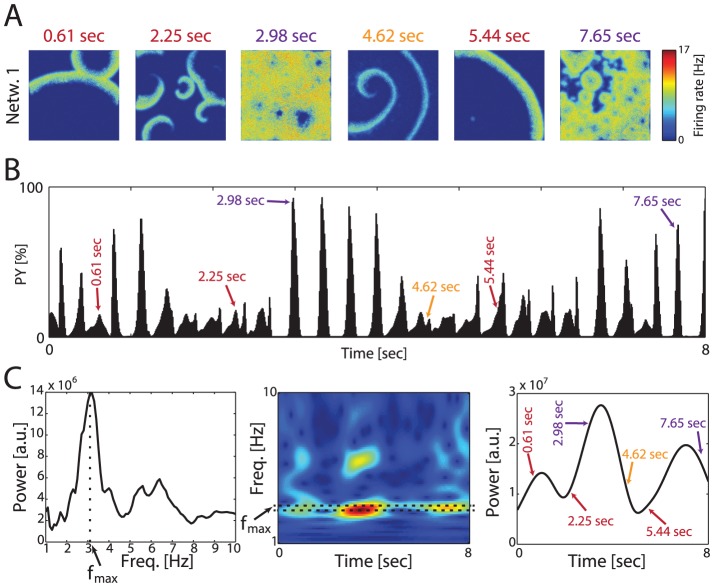
Spontaneous transitions between metastable cortical states. (A) Example simulation with state transitions. Left to right: SP, SP/SW, RF, SW, SP, RF. (B) PY activity profile. Arrows correspond to time snapshots in (A). (C) Spectral power as a function of network state. Left: Time-averaged spectrum exhibits strong peak at endogenous network frequency (f_max_≈3 Hz). Middle: Spectrogram shows pronounced changes in power at f_max_ (dashed box). Right: Time-course of power at f_max_. Colored arrows correspond to time snapshots in (A). RF exhibited highest power at f_max_.

We then analyzed the transitions of individual simulations through these metastable spatio-temporal activity patterns over time (Fig. 4A: LRP delay = 50 msec, P(local) = 0.97, G(LRP) = 0.06, example snapshots of PY activity from a single simulation, time of occurrence indicated in color, SP at 0.61 and 2.25 sec, RF at 2.98 sec, SW at 4.62 sec, SP at 5.44 sec, RF at 7.65 sec; Fig. 4B: PY activity profile with times of example snapshots indicated with arrows). Averaged across time, the spectral power of the network exhibited a peak at the intrinsic oscillation frequency at ∼3 Hz (Fig. 4C, left). However, the spectrogram demonstrated a slow yet pronounced modulation of power at that intrinsic frequency over time (Fig. 4C, middle, epochs with high power in red, dashed lines denote intrinsic network frequency, Fig. 4C, right, power at 3 Hz over time). These fluctuations corresponded to the occurrence of different network states, with RF states being linked to higher power at the intrinsic frequency (Fig. 4C, right). Correspondingly, power at the intrinsic frequency was lower when the system was in SP and SW states. Synaptic depression of the local excitatory coupling played a key role in determining the effect of incoming synaptic activity from the other network (Fig. S4).

### Brain Stimulation Changes Network State

To further understand these different network states, we next applied perturbations to probe the stability of each state. Specifically, we simulated transcranial alternating current stimulation (tACS), which has recently emerged as a promising treatment for psychiatric and neurological illnesses because of its hypothesized ability to selectively manipulate temporal structure of cortical network activity [40], [41], [45], [46]. TACS causes a weak global perturbation of targeted cortical networks due to the low amplitude and broad spatial spread of the weak electric field generated by the scalp stimulation electrodes [47], [48]. Therefore, tACS may be an ideal approach to bias the overall temporal activity structure of interconnected cortical networks.

**Figure 5:**
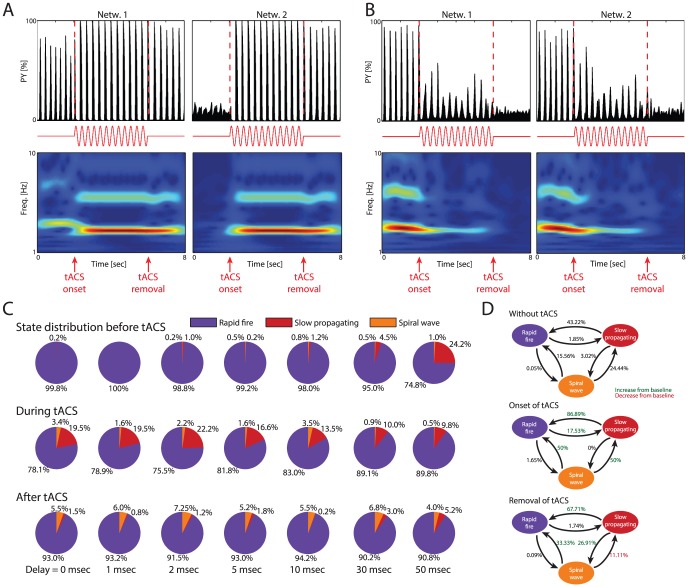
Non-invasive brain stimulation demonstrates multistability of macroscopic activity in interconnected networks. Transcranial alternating current stimulation (tACS) induces outlasting changes in cortical state. (A) Example 1: PY activity plots and spectrograms of simulation receiving tACS. Red: tACS waveform. Network 1 was in RF at onset, which was enhanced by tACS with an outlasting effect on oscillation power. Network 2 began in SW, which was disrupted by tACS, and switched to RF that persisted after removal of tACS. (B) Example 2: Both networks began in RF but were disrupted by the onset of tACS. During tACS the networks exhibited SP with reduced power at 3 Hz compared to pre-onset behavior. After tACS, both networks switched to SW. (C) Percentage of time in each state by delay before, during, and after tACS. During tACS, the amount of spent in SP increased compared to before stimulation and was independent of delay. After tACS, time spent in SP was reduced compared to before tACS (for 10, 30, and 50 msec delays). There was also an increased amount of SW for all delays. (D) Transition probabilities between the network states without tACS (baseline), at the onset of tACS, and at the removal of tACS. Green numbers: Increase from baseline. Red numbers: Decrease from baseline. At the onset of tACS, SW transitions to either RF or SP, while SP and RF had a greater likelihood of transitioning to the other state. Once tACS was removed, SP is maintained less than before stimulation, with a greater chance of transitioning to both SW and RF. SW had a decreased chance of transitioning to SP.

We here used this stimulation modality to probe the dynamic properties of the different activity states that emerged from LRPs with propagation delays. We found that tACS at 3 Hz (close to the endogenous frequency of the individual networks) not only enhanced the synchronization between the two networks but switched the two networks to the fully synchronized, RF state (Fig. 5A, representative simulation, LRP delay 30 msec, P(local) = 0.99, G(LRP) = 0.12; top: activity profiles; middle: stimulation waveform; bottom: spectrograms; see also Movie S4). Network 1 was in RF fire state before tACS onset (distinct peak in the spectrogram at ∼3 Hz) and Network 2 was in SW state (no peak in the spectrogram due to the lack of synchrony within the individual PY network). Importantly, the enhanced, synchronized rhythmic RF activity during stimulation was not limited to the duration of the stimulation but rather outlasted the stimulation. Therefore, the effect of tACS was not just a reflection of the shared input to all PYs but rather represented an outlasting change in activity structure. This “memory” of network activity, in this case during stimulation, is the main feature of a multistable system. The simulated tACS was an effective perturbation, enabling the network to switch to another state (shorter, 1 sec stimulation had the same effect, data not shown).

Interestingly, a small fraction of the simulations did not show this enhancing effect of tACS. Rather, in these cases, tACS switched the networks from RF to either SW or SP states (Fig. 5B, plots same as in Fig. 5A, delay 10 msec, P(local) = 0.97, G(LRP) = 0.06; see also Movie S5). In this simulation, the networks were in synchronized RF state that was switched to SP by tACS and then followed by SW at stimulation removal. To further demonstrate that the state switching by tACS is indeed a consequence of the LRPs, we evaluated models with no LRPs and therefore no communication between the two networks. We found little multistability before and during tACS confirming that LRPs are important for inducing multistable states in cortical networks (Fig. S5).

Given these distinct effects of the same stimulation protocol in different simulations, we determined the relative occurrence of the different states and the state transition probabilities for all simulations (including all propagation delays, fraction of LRPs, and strength of LRPs) as a function of tACS. In the control condition before onset of stimulation (Fig. 5C, top row, Table S3), the majority of simulations exhibited RF behavior with a small fraction demonstrating SP and SW. With increasing propagation delays, the percentage of simulations with SP behavior markedly increased (from 0.2% for 0 msec delay to 24.2% for 50 msec delay). Interestingly, during stimulation (Fig. 5C, middle row), we found the highest fraction of non-RF, and in particular SP, activity patterns in simulations with low LRP propagation delays. As a result, tACS increased the occurrence of the SP state for short propagation delays and decreased the occurrence of SP for longer propagation delays. In further support that such stimulation has a complex effect pattern, we found an increased presence of SW for all delay values after tACS (Fig. 5C, bottom row).

Overall, the state-dependent transition probabilities in the absence of tACS, at tACS onset, and at tACS removal (Fig. 5D) demonstrated that tACS effectively switched activity state, with the most prominent effects being elimination of SP (86.89% transition probability from SP to RF at onset compared to 43.22% in the absence of stimulation) and yet the same stimulation induced a switch from RF to SP in a subset of simulations (17.53% transition probability from RF to SP, compared to 1.8% in the absence of tACS). In turn, if the stimulation succeeded in inducing a transition to RF, the removal of stimulation failed to introduce a state transition back. Specifically, the transition probabilities out of the RF state closely matched the overall transition probabilities in the absence of stimulation (0.09% for RF to SW and 1.74% for RF to SP at stimulation removal in comparison to 0.05% for RF to SW and 1.85% for RF to SP).

We then compared how networks behaved together and found that in the absence of stimulation, both networks were in the RF state for the majority of simulations (Fig. S6, left, 99.66±0.33% for 0 msec delay, 78.68±3.70% for 50 msec delay). With stimulation, there was a small decrease in the percent of time where both PY networks were in RF (81.56±3.27% for 0 msec delay) with the exception of the 50 msec delay simulations (88.81±2.16%, see also Table S4), where the stimulation increased the likelihood of both networks being in RF. In contrast, both SP states were often only found in one of the two networks at a time for delays up to 10 msec (Fig. S6, middle, 0.00±0.00% for 0 msec delay, see also Table S4). For longer delays, simultaneous SP in both networks became much more prominent (17.94±3.50% and 42.74±4.59% for 30 and 50 msec delays, respectively). Similarly, SW never occurred in both PY networks simultaneously before stimulation (0.00%±0.00% for all delay values). Interestingly, during and after stimulation, a subset of simulations exhibited simultaneous SW in both networks, a pattern that never occurred without stimulation (Fig. S6, right). We further examined two simulations that represented peculiarities in our dataset due to their sustained anti-phase locking. Both simulations responded to tACS by switching to (near) zero-lag synchronization that was maintained after stimulation removal (Fig. S7).

### Persistent Oscillation Enhancement by tACS through State Switching

**Figure 6:**
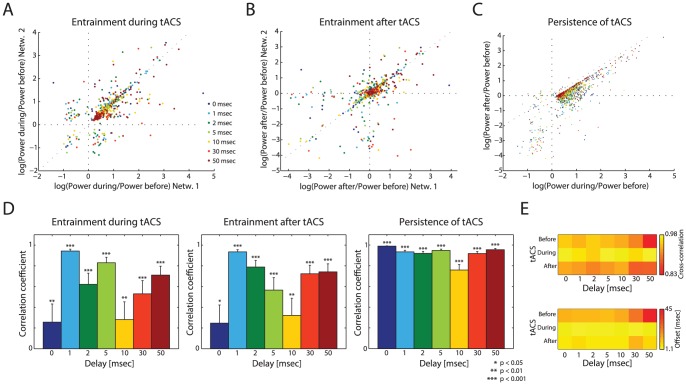
Stimulation enhances endogenous oscillations. (A) Increase in oscillation power by tACS across the two networks. (B) Outlasting effect of stimulation across the two networks. (C) Change in power during tACS versus change in power after tACS for each network over all simulations. Change in power after tACS is correlated to the change in power during tACS, demonstrating an outlasting effect of stimulation. (D) Correlation coefficients by delay for each plot in A–C (significant for all delays, *p*<0.05). (E) Heat map of the mean maximum cross-correlation value and the offset of that value demonstrating the phase shift before, during, and after tACS. The maximum cross-correlation values show that tACS synchronized the two connected networks. The phase offset values show that the network were more phase-synchronized during tACS, an effect that persisted after the removal of tACS. Yellow indicates tighter coupling. Gray dotted lines represent the unity line; error bars represent 95% confidence intervals.

Having established that tACS affects the spatio-temporal activity of two interconnected networks, we next quantified the effect of tACS on the power of the network activity at the stimulation frequency (3 Hz). First, we looked at the effectiveness of tACS to entrain two networks during stimulation by comparing the power during stimulation to the power before stimulation. We found that tACS enhanced the power at 3 Hz of both PY networks during stimulation for most simulations, indicating its ability to entrain networks (Fig. 6A, logarithmic enhancement ratio, 88.1% of all simulations in top right quadrant). The correlation between the enhancement in each of the two networks varied with LRP delay, but with no monotonic relationship between delays (Fig. 6D, left). Next, we analyzed the outlasting effect of tACS after stimulation had stopped. After tACS, the outlasting enhancement was significantly correlated between the two networks, and again with no monotonic relationship between correlation and propagation delay (Fig. 6B, 58.6% of all simulation in top right quadrant and 6D, middle).Thus, tACS can enhance the power of networks at their intrinsic frequencies, an effect that lasts beyond the duration of stimulation. In addition, this enhancement lacks a direct relationship with the values of the propagation delays between the two networks.

To investigate how this outlasting effect of tACS related to the entrainment during stimulation, we compared the enhancement of power at 3 Hz during stimulation to the enhancement of power after stimulation (Fig. 6C, 66.9% of all simulations exhibited enhancement both during and after stimulation). We found that the instantaneous and outlasting effects were tightly correlated, showing that tACS directly increased 3 Hz power (Fig. 6D, right). The cross-correlation peak amplitude and offset, which indicate similarity of behavior and simultaneity of behavior respectively, confirmed these outlasting effects (Fig. 6E, before: 2 sec window before stimulation onset; during: 4 sec of stimulation; after: 2 sec window after stimulation, normalized cross-correlation). With stimulation, the cross-correlation peak was increased (bright yellow), showing that the two networks demonstrate similar behavior during tACS. The phase offset between the two networks was reduced by tACS, an effect that persisted after tACS ended. These effects, together with the outlasting increase in power, show that the two networks were able to sustain a modified network state after tACS. Thus, tACS has an enduring effect on connected networks by entraining the two networks together and increasing their power at the stimulation frequency.

### tACS Disruption and Network Dynamics

**Figure 7:**
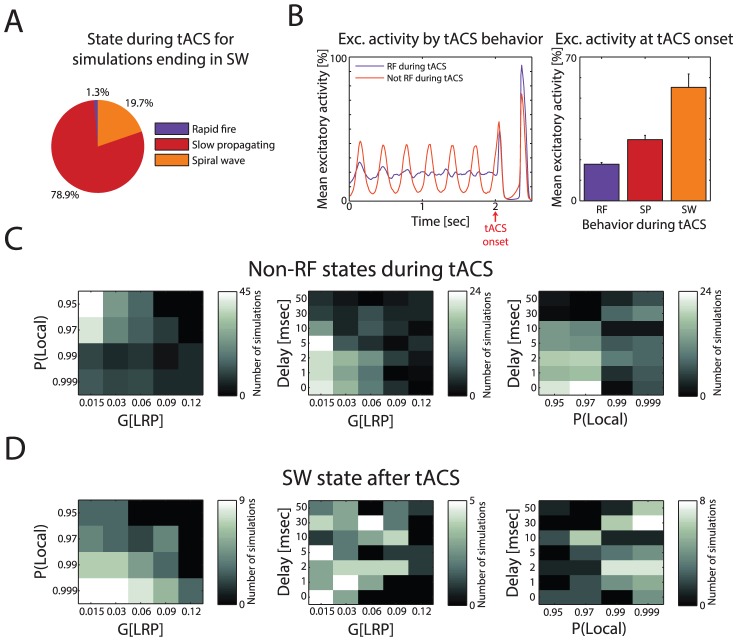
Mechanisms of SW behavior after tACS. (A) Distribution of behavior during tACS for simulations that ended in SW. Most SW simulations were in SP during tACS. (B) Left: Mean PY activity for the first 2.5 seconds of all simulations, grouped by behavior during tACS. Simulations that entered SP or SW during tACS had similar behavior before tACS. Right: Mean PY activity at onset of tACS (t = 2.0). Simulations that entered SP and SW had greater activity at onset than simulations in RF. Error bars indicate s.e.m. (C) Influence of parameters on behavior during tACS. Left: LRP conductance and connectivity. Middle: LRP conductance and delay. Right: LRP connectivity and delay. Weak conductance and high LRP connectivity (low P(local)) predisposed a network against RF during tACS. This effect was enhanced with shorter delays. (D) Influence of parameters on behavior after tACS. Heat maps same as above. Weak LRP conductance and weak connectivity made a network more likely to enter SW after tACS, with no pronounced effect of delay.

Although tACS typically entrained networks to a 3 Hz RF state, occasionally it had an opposite effect by disrupting RF during tACS and causing it to enter SW after tACS. We examined these network dynamics to determine which factors influenced such disruption. Networks that ended in SW after tACS were most often in SP or SW during tACS and only very rarely in RF (Fig. 7A). Consequently, we considered networks in SW and SP during tACS to both be indicators of stimulation-induced state disruption.

When looking at PY activity before tACS, networks in RF during tACS had no specific pattern of activity while networks in SP or SW had a clear temporal structure in their PY activity prior to the onset of tACS (Fig. 7B, left), indicating that the excitatory state of the network was a factor in the response to tACS. The mean PY activity at tACS onset (t = 2.0) showed that networks in SP and SW during tACS had activity levels at onset compared to networks that entered or remained in RF (Fig. 7B, right). This trend suggests that networks in an excited state are more likely to break from RF upon external stimulation. To verify this conjecture, we measured the depression coefficient of each network upon tACS onset (*D*; lower values indicate greater synaptic depression). The depression coefficient was indeed lower for networks that entered SW during tACS, and the normalized variance of *D* was greater for networks in SP or SW during tACS (Fig. S8). Thus, increased synaptic depression, along with a wider variance of depression across the network, predisposed networks towards non-RF behavior, indicating a difficulty in responding to incoming excitation from the other network during a currently- or recently-excited state.

Along with the above described network excitation, however, other factors also facilitated switching to a non-RF state during tACS. Higher LRP connectivity (i.e. lower P(local)) and lower LRP conductance (G(LRP)) both made networks more likely to enter a non-RF state, and these effects were increased with lower delays (Fig. 7C). After tACS, however, networks were more likely to enter SW with lower LRP connectivity and lower conductance, with no clear effect of delay (Fig. 7D). Consequently, lower levels of LRP conductance were more likely to disrupt the RF state while higher levels of LRP conductance generally promoted entrainment and the presence of the RF state. The paradoxical effect of connectivity parameter P(local) indicates that the effect of network topology was altered by stimulation.

The relative prominence of SW after removal of tACS led us to measure the stability of the SW state. We first examined stability of SW in the absence of tACS and found that SW was a metastable state (Fig. S9A). Then we examined longer runs of simulations where at least one network switched to SW after tACS (Fig. S9B). As networks remained in SW for longer periods of time after removal of tACS, the likelihood of them switching from SW decreased, with 28.95% of networks remaining in SW for the entire extended simulation time. Simulations with lower LRP connectivity had longer SW persistence, while LRP conductance and delay had no effect on persistence (Fig. S9C). This effect of connectivity corresponds to that found in Fig. 7D, where less-connected networks are more likely to demonstrate SW behavior. These findings further confirm that SW is a metastable state whose stability is affected by network structure.

### Antiphase tACS Interferes with Synchronization

**Figure 8:**
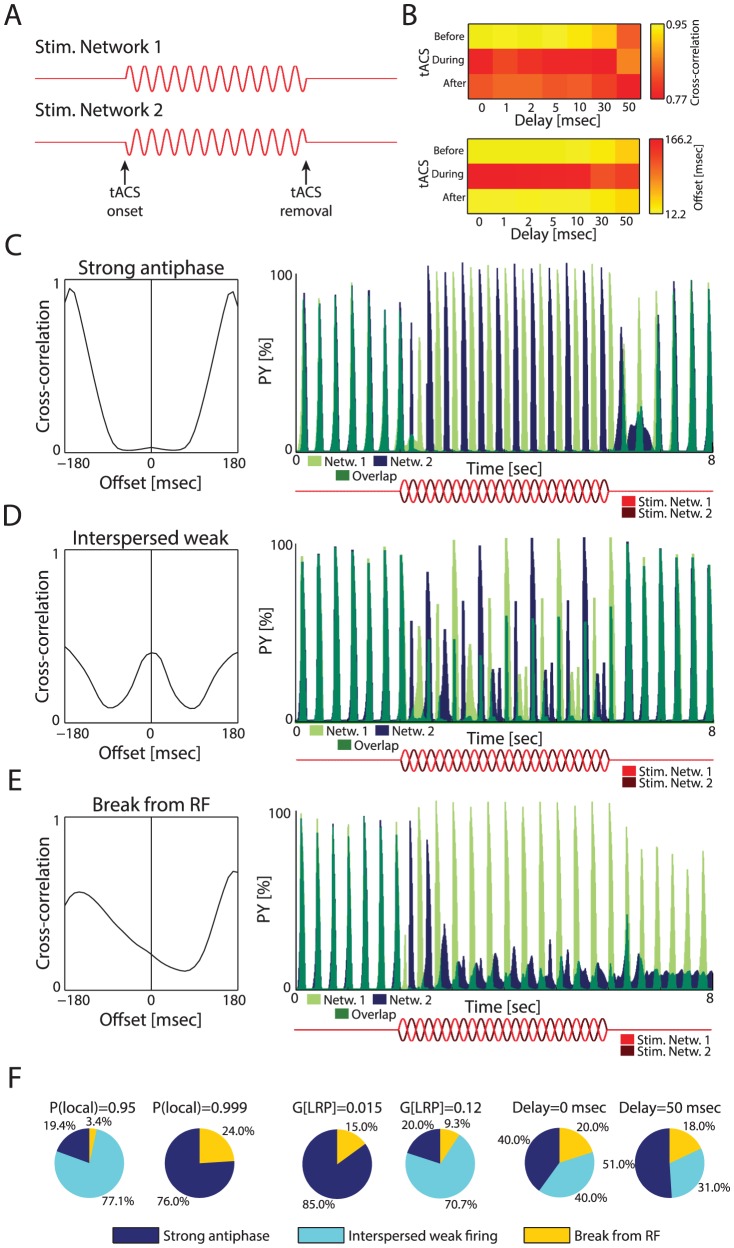
Antiphase tACS induced new behaviors during stimulation. (A) Schematic of antiphase stimulation. (B) Top: Maximum cross-correlation value, indicating similarity of network behavior. Antiphase tACS disrupts network behavior. Bottom: Offset of maximum cross-correlation value indicating phase difference between two networks. Phase difference increased greatly during antiphase tACS but returned to near-baseline levels after removal. (C) Example of strong antiphase tACS behavior. Left: cross-correlogram. Right: PY activity. Both networks fire at 3 Hz during tACS but in antiphase. (D) Example of interspersed weak firing tACS behavior. Networks have strong out-of-phase peaks, but weaker peaks are in phase with the other network. (E) Example of breaking from RF behavior. Network 2 was disrupted by tACS and entered SW after tACS. (F) Effects of parameters on antiphase tACS behavior. Higher connectivity and conductances made interspersed weak firing more likely, while lower LRP connectivity and conductances increased the amount of strong antiphase and breaking from RF behavior. Delays only had a minor effect.

To further probe the mechanisms behind state disruption by tACS, we next simulated antiphase tACS using the same parameters but with the stimulation signal for the two networks phase-shifted by 180 degrees (Fig. 8A). Such stimulation has recently been used in a human tACS study to disrupt phase synchronization yet without direct experimental demonstration of a network effect of out-of-phase tACS [46]. During stimulation, correlation between the activity of the two networks was disrupted, an effect that persisted after removal of tACS (Fig. 8B, top). However, while the networks were out of phase during stimulation, they returned to their original, reduced phase offset after tACS removal (Fig. 8B, bottom). Consequently, antiphase tACS disrupted the dynamics of two interconnected networks but the temporal lag induced by tACS did not persist after tACS removal. When examining the spatio-temporal activity patterns, we found that networks demonstrated three behaviors during antiphase tACS, which were grouped by *k*-means clustering of their cross-correlograms. “Strong antiphase” behavior occurred when the two networks were individually entrained by their respective stimulation (Fig. 8C; Delay = 10 msec, P(local) = 0.99; G(LRP) = 0.06). “Interspersed weak firing” was a result of networks firing in response to both their stimulation as well as the excitation from the other network, resulting in a series of strong and weak UP states (Fig. 8D; Delay = 10 msec; P(local) = 0.99; G(LRP) = 0.09). The third behavior, “breaking from RF”, occurred also with in-phase tACS in the form of SP and SW states (Fig. 8E; Delay = 1 msec, P(local) = 0.99, G(LRP) = 0.09; see Fig. 5B). In this case, one or both of the networks is no longer in RF in response to stimulation.

By examining the effects of parameters on behavior during antiphase tACS, the causes of RF disruption can be more thoroughly uncovered. Higher LRP connectivity (i.e. low P(local)) and higher LRP conductance made interspersed weak firing more likely (Fig. 8F; see Table S5 for all values). This pattern is most likely mediated by the synaptic input from the other network during its UP state. Delays had a minimal effect on behavior during antiphase tACS. Low LRP connectivity most strongly predisposed the networks to break from RF, the converse of what we found during in-phase tACS. Interestingly, the lower LRP connectivity also promoted the persistence of SW after in-phase tACS.

Finally, an interesting behavior arose during antiphase stimulation where the two networks entered a high-frequency (>8 Hz) antiphase state (Fig. S10). This state occurred for all simulations with parameters of Delay = 50 msec, P(local) = 0.95, G(LRP) = 0.12 and for 40% of simulations with Delay = 50 msec, P(local) = 0.95, G(LRP) = 0.09, but no others, and persisted beyond the removal of tACS. This unique behavior further demonstrates the multistability of interconnected cortical networks and the ability of tACS to change network state with outlasting effects.

## Discussion

We used simulations of two large, interconnected cortical networks to study how LRPs that connect the two networks affect the overall macroscopic dynamics. We found that introducing physiologically plausible delays to the LRPs greatly enhanced the repertoire of emergent dynamics, measured not only by synchronization between the two networks but also by the intrinsic spatio-temporal dynamics. Our results therefore suggest small-diameter and unmyelinated projection axons with propagation delays play an important role in enriching the landscape of cortical activity states. This finding contrasts with the traditionally assumed role of long-range connections to enable zero-lag synchrony between different cortical areas [49] and—to our knowledge—for the first time defines a functional role for the large number of slower long-range axons in cortex. In addition, we found that simulated non-invasive brain stimulation can switch the network between these activity states, pointing to its potential applicability for treatment of network-based illnesses.

Our study exclusively utilized computer simulations and therefore has the same caveats as any modeling study. First, the level of abstraction for the model requires consideration. We used computationally efficient, yet biologically plausible model neurons since we were interested in studying the effect of connectivity without confounding the results with the effects of conductance-based, Hodgkin-Huxley-style neuron models, which could model more sophisticated intrinsic cellular dynamics. A reduced model investigating the bifurcations involved in state transitions would provide further insight into network dynamics, although for this study it would reduce the applicability of our findings to the development of novel brain stimulation paradigms. Second, any biologically plausible finding in a computer simulation needs to withstand tests for reasonable robustness to parameter variations. The entire data set presented in this study was based on multiple runs of every simulation with different instantiations of the randomized variables (such as intrinsic excitability and target neurons for global random connections). Third, we believe that the value of most modeling studies can be readily assessed by the type of predictions they make that can then guide subsequent research, whether it be further computational work, wet lab bench studies, or even human preclinical trials. We therefore use the remainder of the discussion section to outline and discuss what we think are the implications and predictions of our results for the study of brain stimulation and network deficits in diseases with altered CNS connectivity such as schizophrenia, autism, and multiple sclerosis.

### Brain Stimulation

Brain stimulation, whether through implanted electrodes such as in deep brain stimulation [50] or through non-invasive application of electric [51] or magnetic fields [52], has established itself as a promising approach for the treatment of a large and growing number of neurological and psychiatric disorders for which only limited pharmacological treatments exist. However, the underlying mechanisms of most of the stimulation paradigms remain hotly debated and little clarity exists with regard to the interaction dynamics between stimulation-induced perturbations and intrinsic network dynamics. We here used simulated transcranial Alternating Current stimulation (tACS) to test if a shared common input to both networks in the form of a weak global perturbation of the PY membrane voltages can synchronize the networks. Based on previous modeling and *in vitro* work [48], [53], [54], we used stimulation waveforms that were matched in frequency to the intrinsic oscillation frequency of the unconnected networks. Interestingly, not only did such 3 Hz sine-wave transcranial current stimulation (tACS) switch the network to a synchronized, rapid fire state, but also—and perhaps more importantly—the network remained in that state at the removal of stimulation in a majority of the simulations. These results suggest that tACS can affect cortical networks by inducing a switch to a qualitatively different, more synchronized network state, which is stable and therefore outlasts the application of the brain stimulation. The amount of time this synchronized state persists after stimulation was not comprehensively mapped. Future work should address which parameters contribute to the persistence of synchronization between two networks; such work can then help to improve the design of non-invasive brain stimulation as a clinical treatment for disorders with impaired synchronization.

Our study suggests that rather than reorganizing synaptic strength, tACS can induce a switch between different macroscopic activity states that are part of a repertoire of cortical states mediated by LRPs with propagation delays. Interestingly, we also found that the same stimulation paradigm had the opposite effect in a (small) subset of simulations where the stimulation reduced the synchronization; these results demonstrate that (1) the ongoing network dynamics (i.e. network state) and the underlying network topology determine the response to brain stimulation and (2) a global stimulus does not necessarily enhance synchronization. Antiphase tACS, a stimulus designed to disrupt synchronization, caused a set of new behaviors during stimulation, but in most cases failed to create antiphase structure between the networks as an outlasting effect. Consequently, the outlasting effects of stimulation are dependent on the phase of stimulation as well as the intrinsic network structure.

As part of a computational model, conclusions drawn from our simulations of tACS are limited by the size of our networks and the fact that each PY receives the same magnitude of stimulation; however, simulated variance of tACS current amplitude has previously been found to have no effect on network response [55]. While it may be necessary to vary the strength of tACS current and electrode size to produce the same effects with patients, our simulations reveal that tACS has the ability to affect network dynamics by introducing periodic excitability into a system. The dependence of the overall effect on current network state at stimulation onset further demonstrates the potential of adaptive, feedback brain stimulation [56], [57] where the stimulation waveform is dynamically adjusted to the ongoing brain activity.

### CNS Diseases with Altered Connectivity

Pathological changes in connectivity in the central nervous system (CNS) are a hallmark of many neurological and psychiatric illnesses. For example, schizophrenia is often called a connectivity disorder due to the findings of aberrations in white matter and lack of functional connectivity in both functional MRI and electroencephalogram (EEG) studies [13], [15], [43], [58]–[69]. We here tested a range of physiologically plausible propagation delays and coupling strengths and found that the occurrence of macroscopic dynamics which lacked synchrony depended on the LRP propagation delays in the presence of slow endogenous rhythmic activity in the individual networks. Therefore, our results predict that disease state and progression can be assayed by determining the structure of global state transitions during awake resting or sleeping, two behavioral states where slow rhythmic activity dominates the spontaneous activity patterns [70], [71]. Furthermore, CNS disorders such as multiple sclerosis [72], where the integrity of the white matter tracts are affected, and epilepsy, which is associated with abnormal cortical oscillations [73]–[75], may lead to similar changes to the landscape of cortical activity states. A spatio-temporal pattern similar to our SP state was recently found to occur in human seizures [76], suggesting that the states in our simulations have biological correlates with the potential to be pathological. Accordingly, these cortical activity states represent a promising target for rational design of (non-)invasive brain stimulation as evaluated in this study.

### Conclusion and Outlook

We used computer simulations of large-scale, interconnected cortical networks in this study and found that long-range projections with physiological delays can play an unanticipated role in generating multistable network dynamics in cortex. Therefore, the so far neglected slow connecting fibers between cortical areas may not be a “flawed design” that prevents large-scale synchronization of cortical areas but rather enables the emergence of additional, qualitatively different network states that likely serve different neural computations. The ability of non-invasive brain stimulation to change these network states points to a promising treatment option for neuropsychiatric disorders involving abnormal connectivity and network dynamics.

## Methods

### Model Neurons

We used the Izhikevich model [6], [77], [78] of pyramidal cells (PYs) and inhibitory interneurons (INs) for the computational simulations in this study. The Izhikevich model provides a very good compromise between biological plausibility and computational efficiency. Each model neuron consists of only two coupled differential equations with four parameters *a*, *b*, *c*, and *d* that determine the intrinsic dynamics. We used an Euler solver with a step width of *Δt* = 0.1 msec such that the update rule at every time-step of the stimulation to compute the new value of the membrane potential *V′* is:
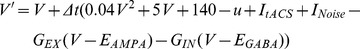


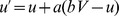


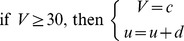


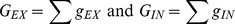
where *V* is the membrane voltage at the previous time-step, *E*_AMPA_ = 0 mV is the excitatory reversal potential (AMPA), *E*_GABA_ = −80 mV is the inhibitory reversal potential (GABA_A_), *G*_EX_ and *G*_IN_ represent the sums of all afferent excitatory (*g*_PY_) and inhibitory (*g*_IN_) conductances, *I*_tACS_ and *I*_Noise_ are current injections to model transcranial alternating current stimulation (tACS) and to cause spontaneous background noise, and *u* is the slow recovery variable.

For PYs, parameters *a* (recovery time-scale) and *b* (recovery sensitivity) were set to 0.02 and 0.2, respectively. We modeled regular spiking, intrinsically bursting, and chattering PY cells by setting the reset potential parameter, *c*, to values from −65 to −50 mV, and the recovery after an action potential, *d*, to values from 6 to 8. All values were drawn from generalized Pareto distributions (*μ* = −50, *σ* = −30, *ξ* = −2, median = −61.26 mV for parameter *c; μ* = 6, *σ* = 4, *ξ* = −2, median = 7.50 for parameter *d*). These distributions helped to bias the parameter values such that regular spiking cells were the most frequent PY cell type. For the INs, the parameters *c* and *d* were set to −65 mV and 2, respectively. To model both fast and low-threshold spiking neurons, parameters *a* and *b* were drawn from uniform distributions (0.02 to 0.1 and 0.2 to 0.25, respectively).

### Model of Synaptic Dynamics

Synapses were model by conductances that were updated with a step in case of a presynaptic action potential and that were subject to exponential decay otherwise. All synapses of a given type were lumped together into a single synapse to increase computational efficiency of the simulations [79]. The respective update rules for the conductances were:
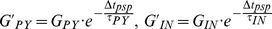
where *G*_EX_ and *G*_IN_ were the corresponding total conductances at the occurrence of the last presynaptic action potential, τ_EX_ = 2 msec and τ_IN_ = 3 msec were the synaptic decay time-constants, and Δ*t*_psp_ was the time elapsed since the last presynaptic action potential. PY-PY connections exhibited short-term synaptic depression [80] with a single depression variable *D* (*D* = 1: no depression, *D* = 0: complete depression) that exhibited an exponential recovery time-course (τ*_D_* = 300 msec). PY-PY synaptic *g*_PY-PY_ strength was calculated as:
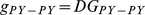
where *G*_PY-PY_ denoted the synaptic strength and *D* was updated for each presynaptic action potential for all PY-PY synapses:
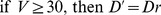
where *r* = 0.6 represented the fraction of synaptic resources available immediately after vesicle release caused by an action potential.

### Network Topology

All simulations in this study consisted of two connected networks. Each network consisted of two layers, a PY network (400×400 model neurons arranged on a two-dimensional grid) and an IN network (200×200 model neurons arranged on a grid). The large number of neurons was motivated by the fact that tACS is likely to act as a global weak perturbation similar to the endogenous electric field [81]. Each PY network exhibited sparse local connectivity where each PY cell connected to a random 30%-subset of 120 cells in its surrounding 11×11 grid of PY cells (*G*_PY-PY_ = 0.06, no autapses). Synaptic inhibition had global random connectivity both for PY-IN excitation (*G*_PY-IN_ = 0.0001, 25 PY-IN connections per PY) and feedback IN-PY inhibition (G_PY-IN_ = 0.0002, 49 IN-PY per IN). The global connectivity scheme for synaptic inhibition was chosen such that inhibition provided an overall activity-dependent reduction of PY firing rate without any extra spatial structure. The synaptic connectivity was chosen such that a 3 Hz endogenous oscillation occurred in the absence of long-range projections (LRPs). LRPs were configured by replacing a defined (small) fraction of local PY-PY connections with excitatory projections to random PYs in the other network (0.1, 1, 3, or 5% of local PY-PY connections). We evaluated the effect of a range of propagation delays for these LRPs (0, 1, 2, 5, 10, 30, and 50 msec).

### Non-synaptic Input Currents

All cells received a current injection *I*_Noise_ that was the sum of (1) a constant current injection ranging from 0 to 1.5 (generalized Pareto distribution with ì = 1, ó = −3, î = −3, median = 0.1895) to create spontaneously firing PYs and (2) a variable current with a random value drawn at every time-step (uniform distribution from 0 to 2 and 0 to 1.5 for PYs and INs, respectively). Non-invasive brain stimulation with transcranial Alternating Current stimulation (tACS) was modeled with a small current injection (*I*_tACS_, amplitude 1.0 corresponding to 10 pA, resulting in average in a membrane voltage depolarization of about 100 µV) into PY cells that are susceptible to applied electric fields because of their elongated somato-dendritic axes [82]–[84].

### Model of Transcranial Alternating Current Stimulation (tACS)

The effect of the electric field resulting from tACS was modeled by injecting a small current into all PYs [81]. The amplitude (10 pA) was chosen such that the corresponding change of the membrane voltage was about 100 µV. INs were not stimulated since they hardly respond to weak electric fields due to their morphology [82]. Stimulation frequency was 3 Hz to match endogenous oscillation frequency of networks.

### Data Analysis

Network activity profiles were determined by the fraction of PY neurons that were firing over time. Both normalized cross-correlations and spectrograms were based on these activity profiles by network. Spectrograms were computed by Wavelet transformation with Morlet wavelets (0.5 to 10 Hz in 0.5 Hz step-width). Macroscopic spatio-temporal activity states were distinguished by the median PY activity peaks (percent PYs firing) in 1 sec bins. Peaks (UP states) were extracted with the Matlab findpeaks function (threshold: 1% of maximum, dead time 50 msec, Mathworks, Natwick, MA). Rapid fire (RF) was assigned to peak values >60% of total number of PYs in the network, slow propagating (SP) was assigned to values 15–60%, and spiral wave (SW) was assigned to values <15%. Relative time spent in different states was determined over all simulations with the two networks considered together. State-dependent transition probabilities were determined for a 1 sec window before stimulation onset, 1 sec after stimulation onset, and last 1 sec window of simulation after stimulation.

### Statistical Analysis

Data are reported as mean±s.e.m. Significance of correlations was determined by corrcoef function in Matlab with 0.05 as significance cut-off.

## Supporting Information

Figure S1 Long-range projections synchronized two cortical networks. (A) Traces of two PYs with LRP conductance of 0 and 0.06. With non-zero LRPs, UP states in PYs synchronize. (B) Power spectrum of PY network activity (red: G(LRP) = 0; blue: G(LRP) = 0.06). LRPs had little effect on overall structure of spectrum but modestly increased peak power.
(PNG)

Figure S2 Comparison of homologous and non-homologous LRPs (zero delay). (A) Activity snapshots. (B) Phase-plane representation. (C) Correlations between the two PY networks. (D–F) Same representation for non-homologous LRPs.
(PNG)

Figure S3 Wider variance of delays stabilized networks. Top: Narrow distribution (mean ±20%). Bottom: Wide distribution (mean ±100%). (A) Frequency of state transitions. (B) State distribution of networks. Wider delays result in fewer transitions and a reduced occurrence of non-RF behavior.
(PNG)

Figure S4 Mechanisms of state transitions. (A) IN activity plots; dashed lines represent example UP states in Network 1. (B) Top and middle: Time snapshots of PY activity in Network 1 and Network 2 for the UP states indicated in (A). Bottom: Synaptic depression variable *D* (*D* = 1: synapses not depressed, *D* = 0 synapses fully depressed). When Network 2 went through a transition towards decreased activity (a, c, e), UP states of Network 1 occurred during a period of strong synaptic depression in Network 2. When Network 2 transitioned towards increased activity (b, d, f, g), the effect of the input of Network 1 was increased due to the reduced synaptic depression allowing more neurons in Network 2 to fire.
(PNG)

Figure S5 Behavior during tACS in unconnected networks. Distribution of behavior for two networks during tACS with no LRPs (P(local) = 1). Left: Before tACS. Middle: During tACS. Right: After tACS. Spiral waves can still be initiated by tACS even without LRPs, but they are not seen before tACS.
(PNG)

Figure S6 Percentage of time both networks were in the same state before, during, and after tACS. Left: tACS equalized the time spent in RF across delays. Middle: tACS also increased the likelihood that both networks were in a SP state. Right: tACS biased networks towards simultaneously being in SW (only seen during and after tACS). Values are normalized by the percentage of time spent in each state overall.
(PNG)

Figure S7 tACS abolished antiphase synchronization. (A) Example of slow antiphase coupling. Upper left: Cross-correlograms between networks before, during, and after tACS. Lower left: Network 2 displayed a state transition before entraining with Network 1 during stimulation. Right: Increased power at 3 Hz in both networks due to tACS. (B) Example of fast antiphase coupling suppressed by tACS. Same plots as in (A).
(PNG)

Figure S8 Synaptic depression influenced tACS behavior. Left: Depression coefficient at onset of tACS, grouped by behavior during tACS. Lower values indicate more synaptic depression; higher values indicate less synaptic depression. Networks entering SW during tACS had more strongly depressed networks than networks entering RF or SP. Right: Standard deviation of the depression coefficient normalized by the mean. Lower variance of depression correlates with stronger entrainment to tACS.
(PNG)

Figure S9 Persistence of spiral waves. (A) The three simulations that had constant SW behavior during original simulations, extended for another 7 seconds. One simulation (top) remained in SW except for a brief switch to SP, while the other two simulations (middle and bottom) stayed in SW for the entire time. (B) Persistence of SW in networks that ended with SW after tACS. Simulations ran for another 7 seconds. X-axis indicates the number of seconds SW persisted in the extended period. Many networks leave SW, but 22 networks (28.95%) remain in SW for the entire extended period. (C) Effects of parameters on SW persistence. Lower connectivity (left) correlates with longer persistence of SW. Conductance (middle) and delays (right) have no effect.
(PNG)

Figure S10 Antiphase induction of high-frequency behavior post-tACS. (A) PY activity during antiphase tACS. During stimulation, the network switches from in-phase ∼3 Hz firing to antiphase firing at 8.6 Hz, persisting upon removal of tACS. (B) Spectrogram shows change from 3 Hz firing to rapid high-frequency firing in both networks.
(PNG)

Movie S1 Example of rapid fire state. PY activity for two networks is shown in color, indicating the instantaneous firing rate. Most of each network became excited rapidly. Parameters: Delay = 0 msec, P(local) = 0.99, G(LRP) = 0.06.
(AVI)

Movie S2 Example of slow propagating state. PY activity for two networks in SP for entire simulation. PY activity spread through the network by moving to proximal areas. Parameters: Delay = 50 msec, P(local) = 0.95, G(LRP) = 0.03.
(AVI)

Movie S3 Example of spiral wave state. Network 1 was in SW for entire simulation while Network 2 was in RF for whole simulation. Spiral wave began with central rotor from which PY activity propagated, forming spiral pattern. Parameters: Delay = 10 msec, P(local) = 0.99, G(LRP) = 0.015.
(AVI)

Movie S4 tACS entrained networks. Simulation presented in Fig. 5A. Network 1 began in RF while Network 2 demonstrated SW. Upon tACS (bottom, red) the two networks entrained to tACS and Network 2 switched to RF. Both networks remained in RF after removal of tACS. Parameters: Delay = 30 msec, P(local) = 0.99, G(LRP) = 0.12.
(AVI)

Movie S5 tACS disrupted networks. Simulation presented in Fig. 5B. Both networks began in RF state. Upon tACS (bottom, red) RF was disrupted, creating a mixed SP/SW state. The networks settled in SW upon removal of tACS. Parameters: Delay = 10 msec, P(local) = 0.97, G(LRP) = 0.06.
(AVI)

Table S1 Multistability as a function of propagation delays (Figure 3D and 3E).
(XLSX)

Table S2 Distributed delays reduced state transitions (Figure S3).
(XLSX)

Table S3 Correlation (R^2^) of dynamics between the two interconnected networks (Figure 6D).
(XLSX)

Table S4 Modulation of state dynamics by simulated tACS as a function of propagation delays (Figure S6C).
(XLSX)

Table S5 Effects of simulation parameters on tACS behavior.
(XLSX)

Text S1 Synaptic depression contributes to state dynamics. An analysis of the effect of synaptic depression within a network on its response to input from the network connected to it.
(DOCX)
