# Supplementary figures and images for: Emergence of Metastable State Dynamics in Interconnected Cortical Networks with Propagation Delays
Source: PLoS Comput Biol. 2013 Oct 24;9(10):e1003304. doi: 10.1371/journal.pcbi.1003304 (PMC3812055; doi:10.1371/journal.pcbi.1003304)

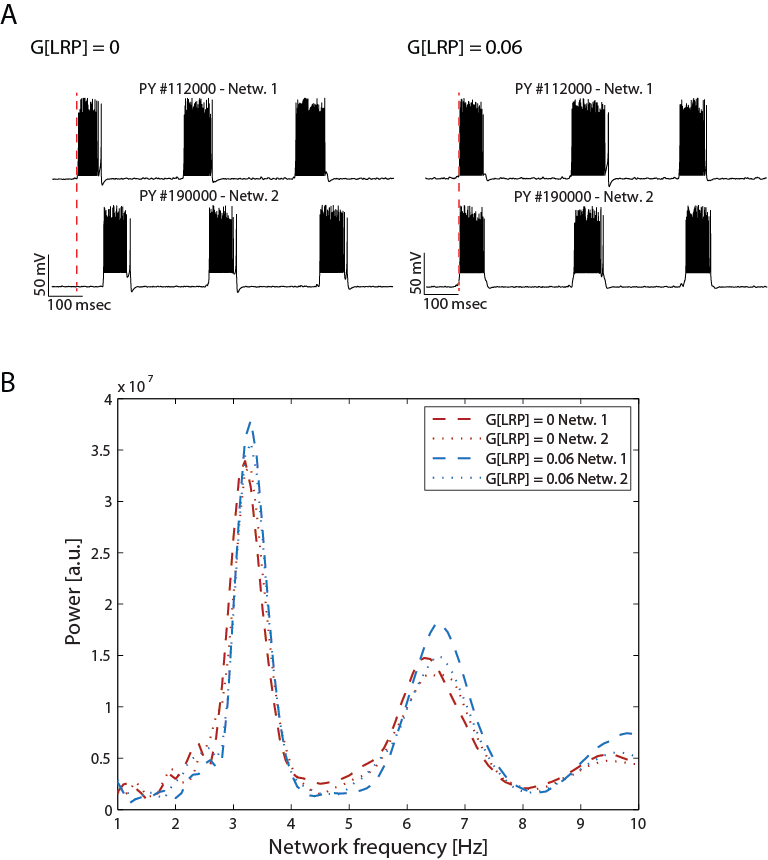

Supplement: Figure S1 — Long-range projections synchronized two cortical networks. (A) Traces of two PYs with LRP conductance of 0 and 0.06. With non-zero LRPs, UP states in PYs synchronize. (B) Power spectrum of PY network activity (red: G(LRP) = 0; blue: G(LRP) = 0.06). LRPs had little effect on overall structure of spectrum but modestly increased peak power. (PNG) [file pcbi.1003304.s001.png]

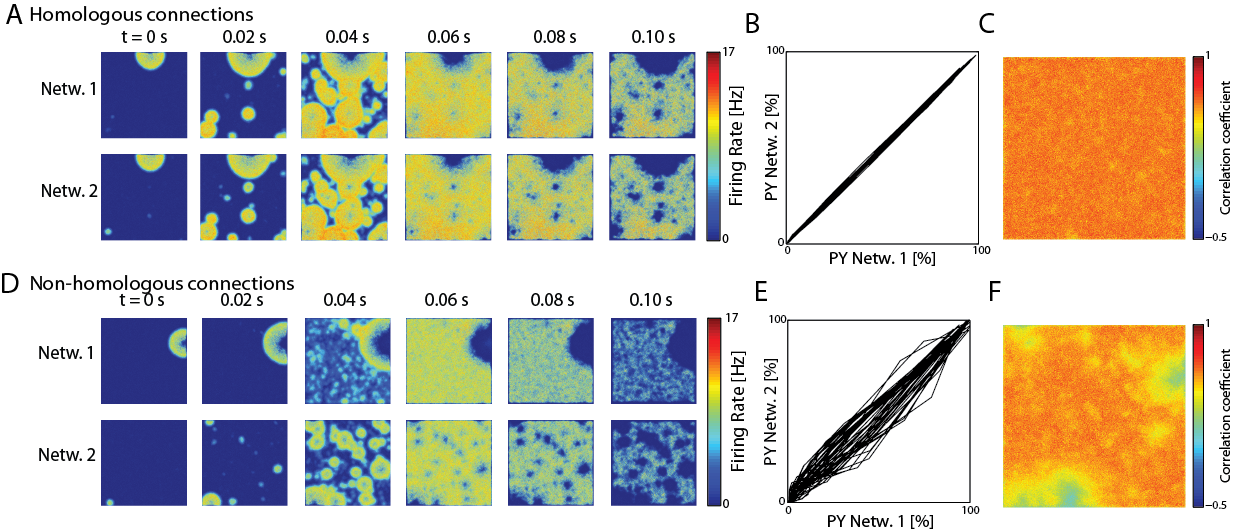

Supplement: Figure S2 — Comparison of homologous and non-homologous LRPs (zero delay). (A) Activity snapshots. (B) Phase-plane representation. (C) Correlations between the two PY networks. (D–F) Same representation for non-homologous LRPs. (PNG) [file pcbi.1003304.s002.png]

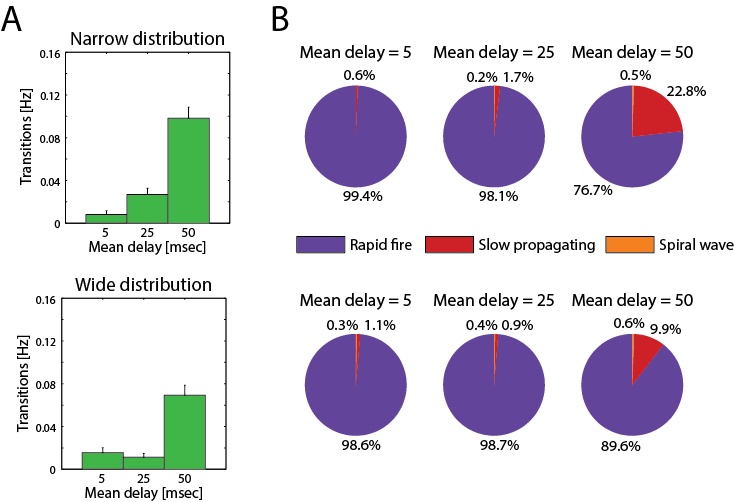

Supplement: Figure S3 — Wider variance of delays stabilized networks. Top: Narrow distribution (mean ±20%). Bottom: Wide distribution (mean ±100%). (A) Frequency of state transitions. (B) State distribution of networks. Wider delays result in fewer transitions and a reduced occurrence of non-RF behavior. (PNG) [file pcbi.1003304.s003.png]

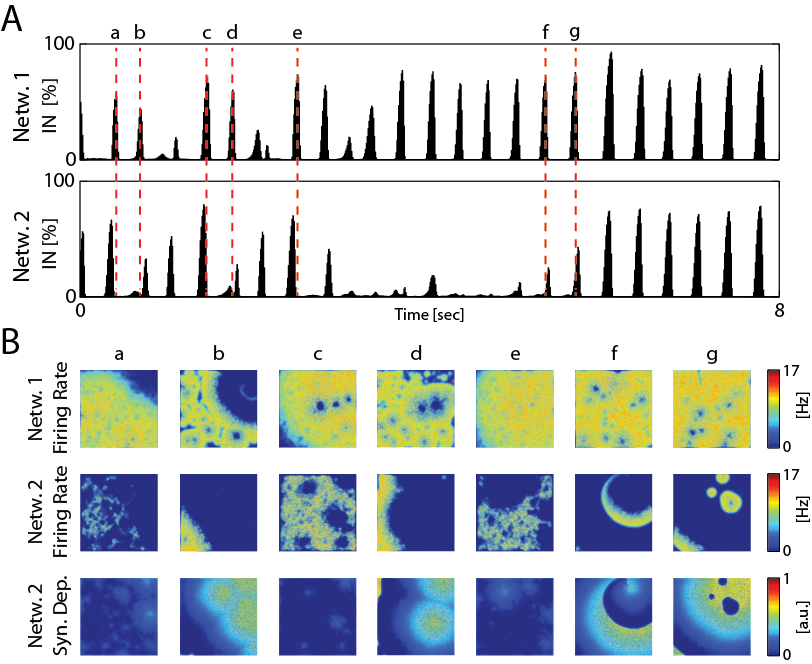

Supplement: Figure S4 — Mechanisms of state transitions. (A) IN activity plots; dashed lines represent example UP states in Network 1. (B) Top and middle: Time snapshots of PY activity in Network 1 and Network 2 for the UP states indicated in (A). Bottom: Synaptic depression variable D (D = 1: synapses not depressed, D = 0 synapses fully depressed). When Network 2 went through a transition towards decreased activity (a, c, e), UP states of Network 1 occurred during a period of strong synaptic depression in Network 2. When Network 2 transitioned towards increased activity (b, d, f, g), the effect of the input of Network 1 was increased due to the reduced synaptic depression allowing more neurons in Network 2 to fire. (PNG) [file pcbi.1003304.s004.png]

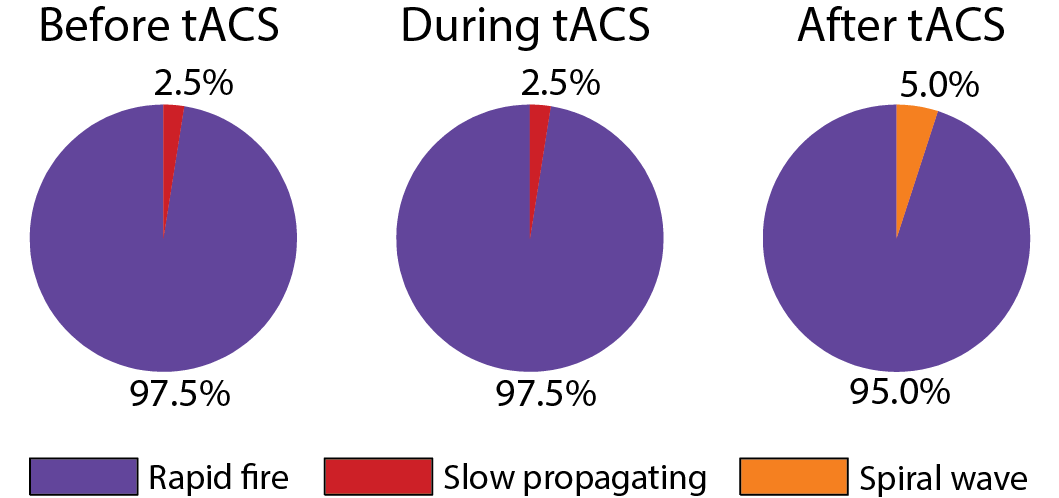

Supplement: Figure S5 — Behavior during tACS in unconnected networks. Distribution of behavior for two networks during tACS with no LRPs (P(local) = 1). Left: Before tACS. Middle: During tACS. Right: After tACS. Spiral waves can still be initiated by tACS even without LRPs, but they are not seen before tACS. (PNG) [file pcbi.1003304.s005.png]

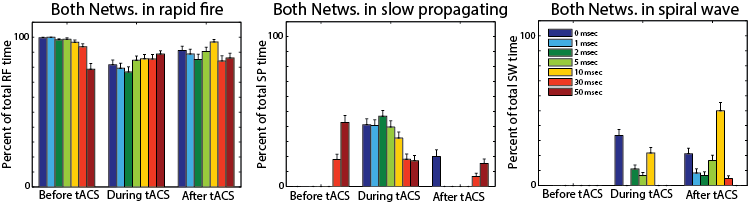

Supplement: Figure S6 — Percentage of time both networks were in the same state before, during, and after tACS. Left: tACS equalized the time spent in RF across delays. Middle: tACS also increased the likelihood that both networks were in a SP state. Right: tACS biased networks towards simultaneously being in SW (only seen during and after tACS). Values are normalized by the percentage of time spent in each state overall. (PNG) [file pcbi.1003304.s006.png]

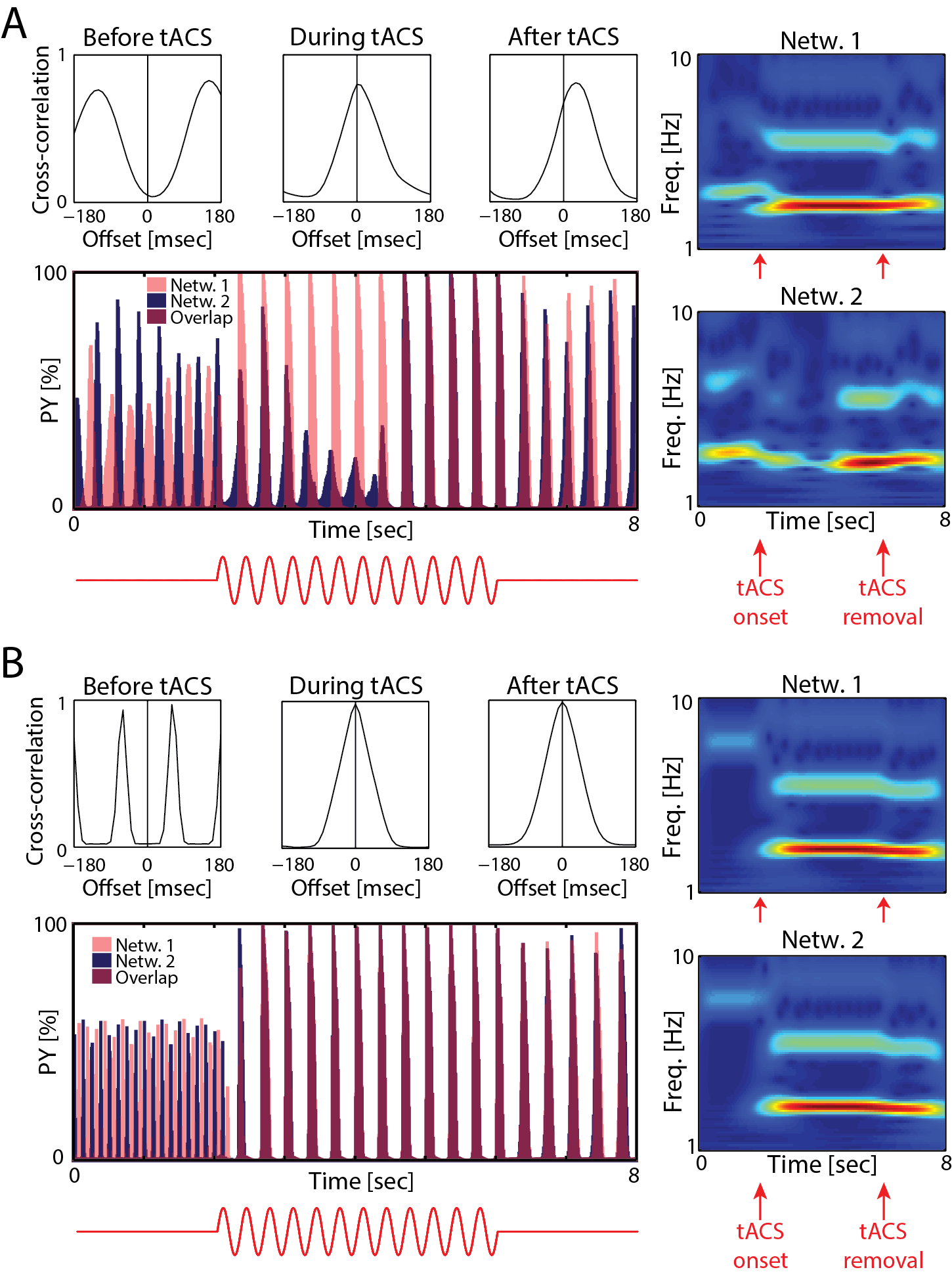

Supplement: Figure S7 — tACS abolished antiphase synchronization. (A) Example of slow antiphase coupling. Upper left: Cross-correlograms between networks before, during, and after tACS. Lower left: Network 2 displayed a state transition before entraining with Network 1 during stimulation. Right: Increased power at 3 Hz in both networks due to tACS. (B) Example of fast antiphase coupling suppressed by tACS. Same plots as in (A). (PNG) [file pcbi.1003304.s007.png]

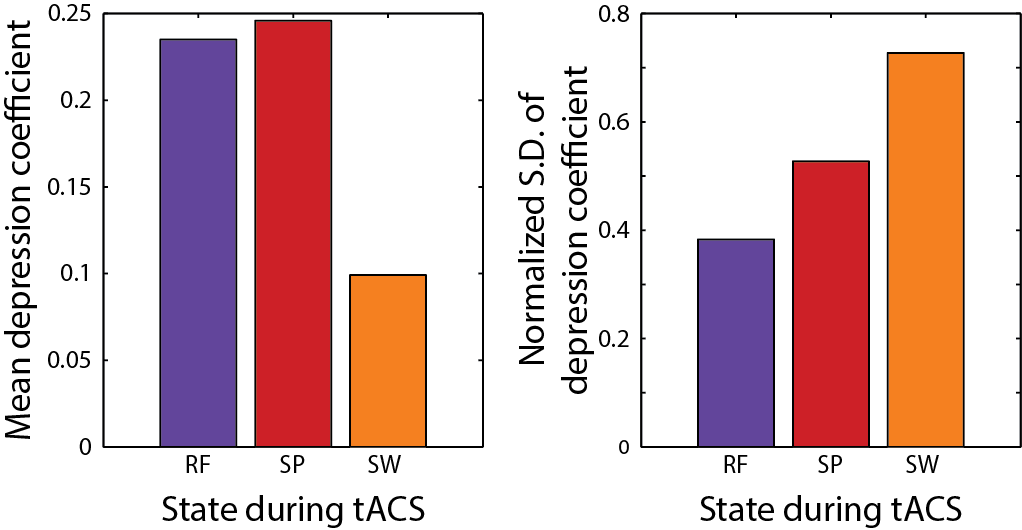

Supplement: Figure S8 — Synaptic depression influenced tACS behavior. Left: Depression coefficient at onset of tACS, grouped by behavior during tACS. Lower values indicate more synaptic depression; higher values indicate less synaptic depression. Networks entering SW during tACS had more strongly depressed networks than networks entering RF or SP. Right: Standard deviation of the depression coefficient normalized by the mean. Lower variance of depression correlates with stronger entrainment to tACS. (PNG) [file pcbi.1003304.s008.png]

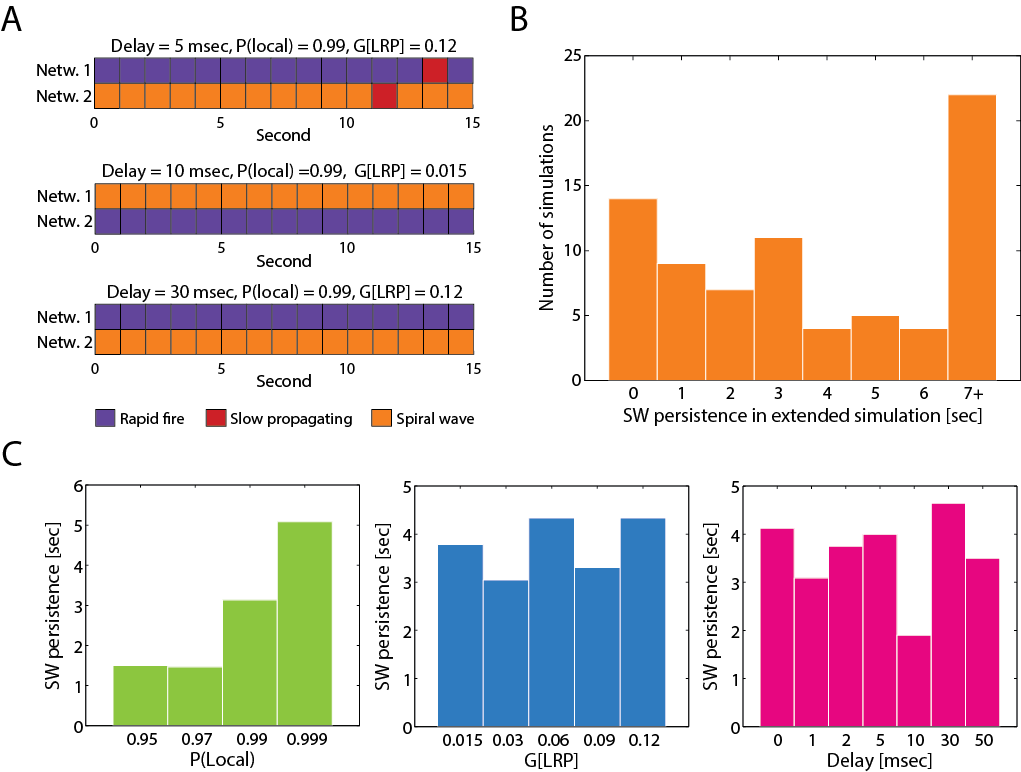

Supplement: Figure S9 — Persistence of spiral waves. (A) The three simulations that had constant SW behavior during original simulations, extended for another 7 seconds. One simulation (top) remained in SW except for a brief switch to SP, while the other two simulations (middle and bottom) stayed in SW for the entire time. (B) Persistence of SW in networks that ended with SW after tACS. Simulations ran for another 7 seconds. X-axis indicates the number of seconds SW persisted in the extended period. Many networks leave SW, but 22 networks (28.95%) remain in SW for the entire extended period. (C) Effects of parameters on SW persistence. Lower connectivity (left) correlates with longer persistence of SW. Conductance (middle) and delays (right) have no effect. (PNG) [file pcbi.1003304.s009.png]

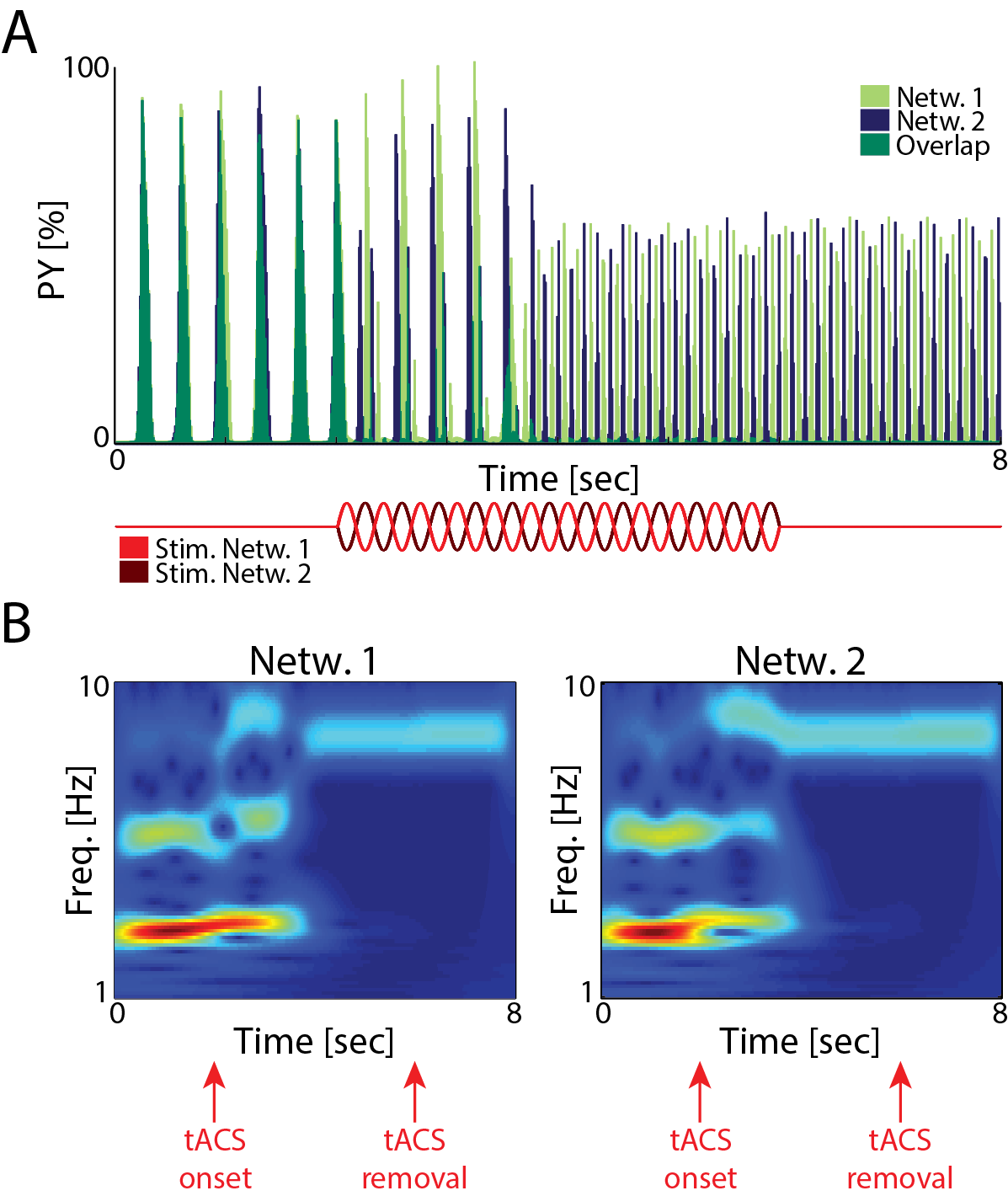

Supplement: Figure S10 — Antiphase induction of high-frequency behavior post-tACS. (A) PY activity during antiphase tACS. During stimulation, the network switches from in-phase ∼3 Hz firing to antiphase firing at 8.6 Hz, persisting upon removal of tACS. (B) Spectrogram shows change from 3 Hz firing to rapid high-frequency firing in both networks. (PNG) [file pcbi.1003304.s010.png]
