# Supplementary material for: Emergence of Metastable State Dynamics in Interconnected Cortical Networks with Propagation Delays
Source: PLoS Comput Biol. 2013 Oct 24;9(10):e1003304. doi: 10.1371/journal.pcbi.1003304 (PMC3812055; doi:10.1371/journal.pcbi.1003304)
Supplement: Text S1 — Synaptic depression contributes to state dynamics. An analysis of the effect of synaptic depression within a network on its response to input from the network connected to it. (DOCX) [file pcbi.1003304.s021.docx]

**Text S1**

Kutchko and Frohlich 2013

*Synaptic Depression Contributes to State Dynamics*

We examined the mechanism that mediated state transitions; we found that the relative time (oscillation phase) at which a network received input from the other network by LRPs was instrumental in determining whether a state transition occurred (Fig. S3A, IN activity profile that reflects overall network state due to its global PY excitatory inputs). UP states that occurred in Network 1 briefly *after* an UP state in Network 2 had limited effect on Network 2 (UP states a, c, e). UP states with some overlap with activity in Network 2 had an intermediate effect (UP states b and d) and UP states that occurred during a DOWN state in Network 2 had the greatest effect (UP states f, g). Not only did these UP states trigger a full-blown UP state in Network 2 but also triggered a state transition from SW to RF. Inspecting the matched time snapshots of PY activity (Fig. S3B, top two rows, time-snap shots of Network 2) and the degree to which the local excitatory synapses were depressed in Network 2 (Fig. S3B, bottom row, a value of 1 corresponded to no synaptic depression where a value of 0 corresponded to a completely depressed synapse) revealed that the effect of Network 1 on Network 2 depended on the degree to which the local synapses were depressed in Network 2. UP states that had an intermediate effect (b, d) all occurred during times with reduced synaptic depression (0.24±0.17 and 0.20±0.16, mean±std). UP states that had no effect (a,c,e) occurred at times of uniformly strong synaptic depression (0.062±0.059, 0.024±0.033, 0.047±0.053, respectively). The two UP states that induced synchronization between the two networks (f,g) had similar mean synaptic depression to the UP states that had an intermediate effect (0.22±0.15 and 0.27±0.18) that enabled the transition dynamics. Therefore, synaptic depression of local excitatory synapses played a key role in enabling the occurrence of state transitions in the overall network.
